# Tinea Manuum Caused by Trichophyton benhamiae Following Exposure to a Pet Guinea Pig in the United States

**DOI:** 10.7759/cureus.110255

**Published:** 2026-06-04

**Authors:** Brailyn Weber, Bret Andersen, Maggie Moses

**Affiliations:** 1 Dermatology, University of North Dakota, Fargo, USA; 2 Dermatology, Marshfield Clinic Health System, Marshfield, USA

**Keywords:** case report, dermatology, dermatophyte, guinea pig, tinea manuum, trichophyton benhamiae

## Abstract

Tinea manuum may result from direct contact with animals infected with zoophilic dermatophytes. We present a case of tinea manuum in a 32-year-old woman caused by *Trichophyton benhamiae*, an emerging fungal pathogen frequently associated with guinea pig exposure. This case highlights a potentially unique pattern of zoonotic transmission, encourages clinicians to consider *T. benhamiae* in the differential diagnosis of inflammatory tinea manuum, and underscores the importance of advanced diagnostic methods for accurate fungal identification.

## Introduction

Tinea manuum is a superficial fungal infection of the hands that typically manifests as an erythematous, scaly annular rash involving the palms, dorsum, or interdigital spaces. The most common mode of transmission is autoinoculation from concomitant onychomycosis or tinea pedis [1]. In these cases, infection with anthropophilic dermatophytes is characteristic, with *Trichophyton rubrum* being the most common causative organism [2]. Less frequently, tinea manuum may be acquired through direct contact with animals infected with zoophilic dermatophytes, including *Microsporum canis*, *Trichophyton mentagrophytes*, and *Trichophyton verrucosum* [3]. To our knowledge, we report the first documented case of tinea manuum caused by *Trichophyton benhamiae* in the United States. This fungus is frequently associated with guinea pig exposure and has emerged as a zoonotic dermatophyte, with cases reported in Europe, Asia, and South America. Guinea pigs are considered the primary animal reservoir and may remain asymptomatic despite infection, highlighting the diagnostic challenges and risk of unrecognized transmission.

## Case presentation

A 32-year-old woman presented to the dermatology clinic for evaluation of a red, scaly, and intensely pruritic rash localized to the palmar and dorsal aspects of her right distal hand and third through fifth fingers. She also reported a more chronic history of small fluid-filled vesicles involving the palmar aspects of both hands and fingers. The rash had gradually worsened over the preceding month and was associated with pain, swelling, and difficulty flexing and extending the affected fingers.

Prior to presentation, the patient had been evaluated at an urgent care clinic on several occasions, where she was diagnosed with dyshidrotic eczema with secondary impetiginization. Previous treatments included over-the-counter hydrocortisone, triamcinolone 0.025% lotion, clobetasol 0.05% cream, and a seven-day course of oral cephalexin. The patient reported worsening of the hand rash after using topical corticosteroids, particularly clobetasol.

The patient worked in an office-based role involving frequent typing and denied wet work or other occupational hand exposures. Additional history revealed that she owned two pet guinea pigs: one for the past seven years and a second acquired approximately six months before the onset of her rash. She reported occasional direct contact with and handling of both animals. The newer guinea pig had recently developed an area of hair loss with overlying scale on the posterior aspect of its back, which the patient had attributed to grooming behavior by the older guinea pig. She denied close contact with similar symptoms and had no personal history of dermatologic or atopic disease.

**Figure 1:**
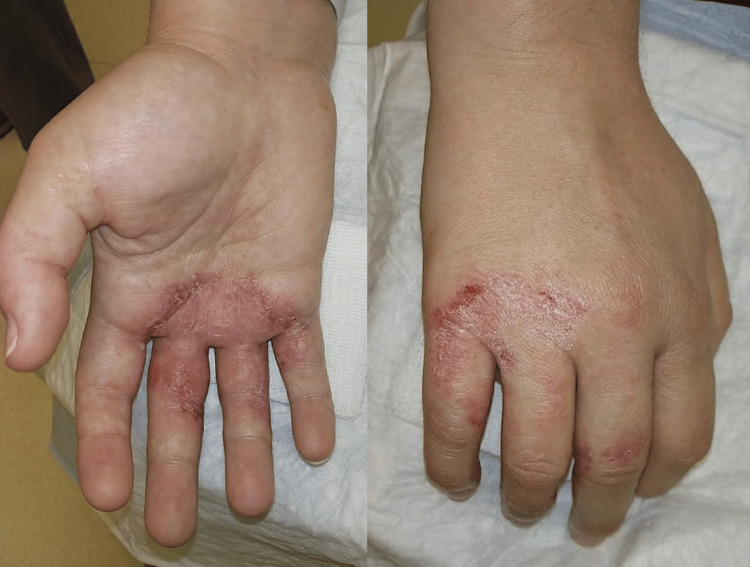
Annular, erythematous, scaly plaque with fissuring and peripheral pustules on the right distal palmar surface extending onto the distal dorsal hand and dorsal aspect of the proximal 3rd-5th fingers.

Physical examination revealed an annular, erythematous, scaly plaque with fissuring and peripheral pustules on the distal palmar surface of the right hand, extending onto the distal dorsal hand and the dorsal aspects of the proximal third through fifth fingers (Figure 1). 

**Figure 2:**
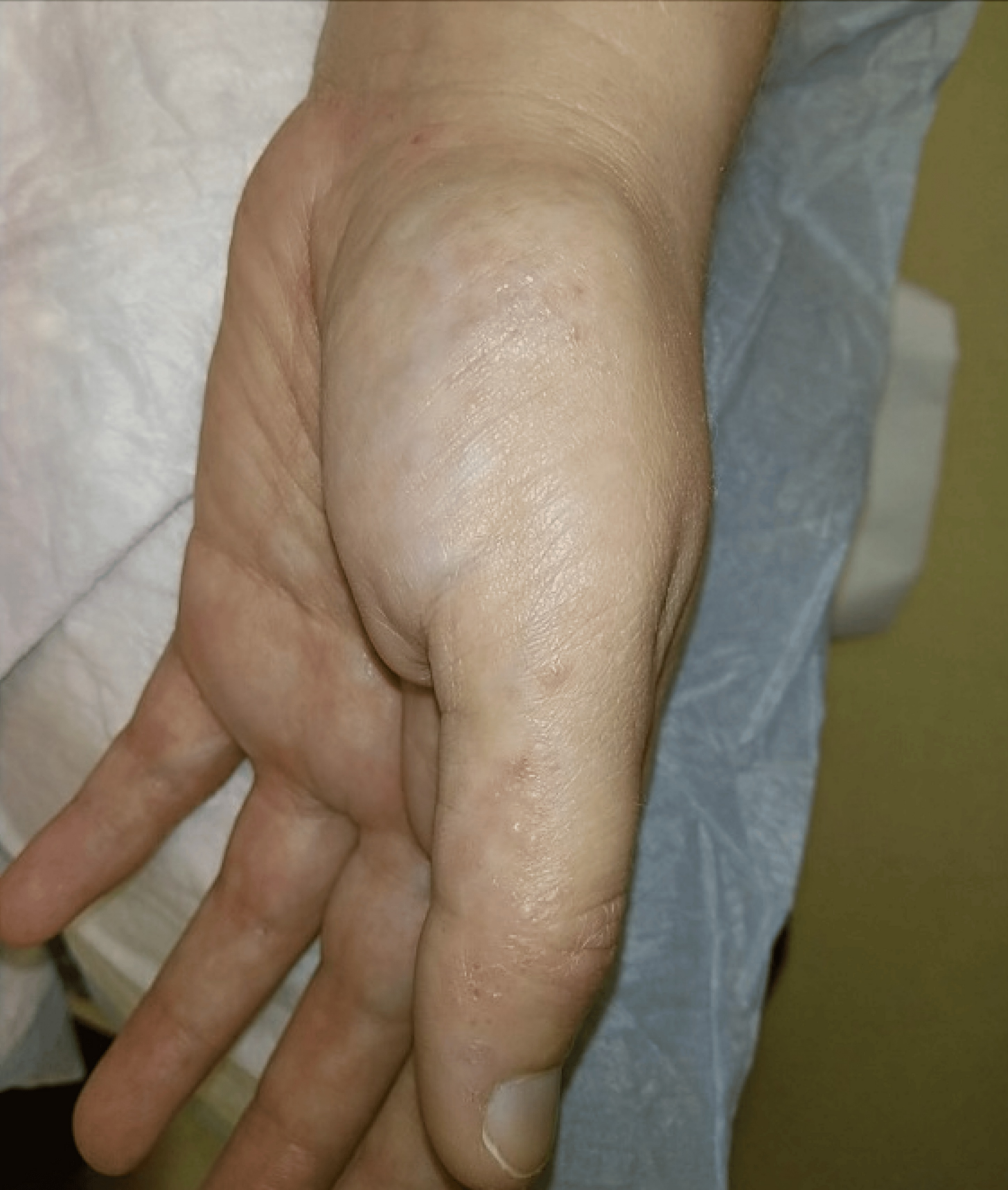
Scattered erythematous papules and vesicles on the palmar surface of the left hand.

Additionally, multiple scattered erythematous papules and vesicles were present on the palmar surfaces of both hands and fingers (Figure 2). The initial differential diagnosis included tinea manuum, dyshidrotic eczema, and allergic contact dermatitis. 

**Figure 3:**
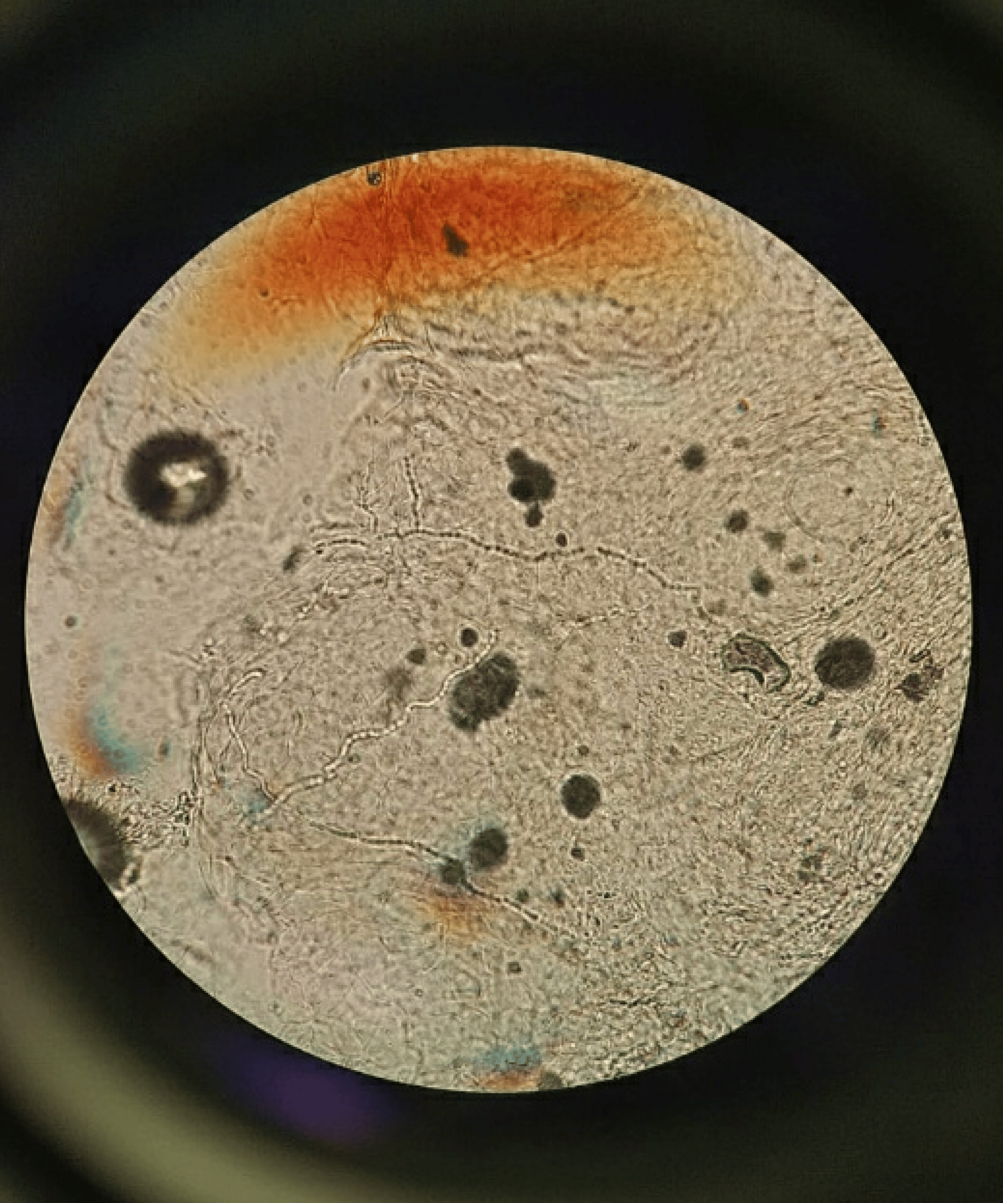
Septate hyphae and arthroconidia visualized on KOH preparation of scale and pustular exudate from the patient’s right hand.

A potassium hydroxide (KOH) preparation of scale and pustular exudate obtained from the patient's right hand revealed septate hyphae and arthroconidia (Figure 3).

Subsequently, a dermatophyte culture obtained from scale and a fungal culture obtained from a punch biopsy specimen were performed. Phenotypic findings and polymerase chain reaction (PCR) were used for species identification, ultimately confirming a diagnosis of tinea manuum caused by *T. benhamiae*. In addition, the patient's significant pain, swelling, and difficulty with finger flexion and extension raised concern for a deep soft tissue infection or osteomyelitis. A radiograph of the right hand was obtained and was negative for any bony or soft tissue abnormalities.

Based on these findings, the patient was started on oral terbinafine 250 mg once daily for six weeks. She was also instructed to apply butenafine 1% cream to the affected areas twice daily for four weeks. Several weeks after initiating terbinafine therapy, the patient developed a generalized morbilliform rash. Terbinafine was subsequently discontinued, and she was transitioned to oral itraconazole 200 mg twice daily for four weeks. The patient responded well to this treatment.

## Discussion

Tinea manuum is a superficial fungal infection of the hands that typically presents with erythematous, scaly patches or plaques, which may be annular, and is often associated with pruritus. Autoinoculation from concomitant onychomycosis or tinea pedis is the most common mode of transmission and occurs when patients scratch infected skin on their feet, producing the classic "two feet-one hand syndrome" [1,2]. Trichophyton rubrum, an anthropophilic dermatophyte, is the most commonly implicated pathogen in these cases [2]. Less frequently, tinea manuum may result from direct contact with animals infected with zoophilic dermatophytes. Common etiologic agents include *M. canis*, *T. mentagrophytes*, and *T. verrucosum* [3]. In such cases, lesions are often more inflammatory and may be accompanied by edema, vesicles, or pustules [4]. Although less common, tinea manuum may also be caused by nondermatophyte fungi, which are often more difficult to manage and respond less reliably to antifungal therapy [2].

To our knowledge, we report the first documented case of tinea manuum caused by *T. benhamiae*, likely associated with guinea pig exposure, in the United States. *T. benhamiae* is a zoophilic dermatophyte that has recently emerged as a cause of human fungal infections in many countries outside the United States, including Japan [5], China [6], Brazil [7], Taiwan [8], Spain [9,10], Portugal [11], Iran [12], and Argentina [13]. To date, only one case of *T. benhamiae* infection has been reported in the United States, involving a child with tinea faciei [14]. Guinea pigs are the primary animal reservoir and may appear clinically normal despite infection. In a 2017 study of 15 German pet shops, *T. benhamiae* was identified in more than 90% of tested animals (53 of 59), although only 9% exhibited visible signs of tinea [15]. Case reports have also documented transmission from animals other than guinea pigs, including cats, dogs, rabbits, foxes, and other small rodents [5-14].

Marked inflammation at presentation is a common feature of *T. benhamiae* dermatophytosis, as observed in our patient. The inflammation was severe enough to impair finger flexion and extension, prompting radiographic evaluation of the hand. Several published cases have similarly described significant inflammation associated with *T. benhamiae* infection. Wang and Sun [8] reported a case of blepharociliaris associated with eyelid swelling and eyelash loss, while Arias et al. [14] described a case of tinea faciei in a seven-year-old child who presented with a fever of 38.5°C. It is also important to consider the potential predisposition to infection in patients with skin barrier dysfunction. In our patient, the infection may have been facilitated by underlying dyshidrotic eczema, as suggested by the deep-seated vesicles observed on physical examination. Notably, her symptoms worsened with topical clobetasol treatment, indicating that dyshidrotic eczema alone did not account for the clinical presentation. Systemic diseases may also increase susceptibility to infection, as demonstrated in the case reported by Wang and Sun [8], in which the patient had poorly controlled type 1 diabetes mellitus.

KOH preparations of specimens infected with *T. benhamiae* commonly reveal septate hyphae and arthroconidia; however, these findings may be indistinguishable from those of other dermatophytes. Definitive identification therefore requires fungal culture and/or molecular diagnostic techniques. Fungal cultures classically demonstrate flat, velvety, beige-yellow colonies with radiating edges, while species confirmation may be achieved through PCR, sequencing, or mass spectrometry [16].

Treatment of *T. benhamiae* infection generally consists of topical antifungal agents effective against dermatophytes, such as azoles and allylamines. For extensive infections, immunosuppressed patients, or infections involving hair-bearing areas, systemic therapy is indicated. In a recent study by Shamsizadeh et al. [17], *T. benhamiae* demonstrated the greatest susceptibility to terbinafine based on minimum inhibitory concentration testing, consistent with its use in most reported cases. Other systemic treatment options include griseofulvin, itraconazole, and fluconazole, although they may demonstrate lower in vitro activity.

This case report has several limitations. First, microbiologic confirmation of *T. benhamiae* infection in the patient's pet guinea pig was not obtained, limiting the ability to definitively establish a zoonotic source of infection. Additionally, as a single-case report, the findings are limited in their ability to establish causality or be generalized to larger populations.

## Conclusions

Altogether, this report describes a case of tinea manuum caused by *T. benhamiae*, an emerging dermatophyte commonly associated with exposure to guinea pigs. To our knowledge, this represents the first reported case of tinea manuum due to this pathogen in the United States. Clinicians should be aware of the reported zoonotic transmission pattern associated with guinea pigs and include *T. benhamiae* in the differential diagnosis of inflammatory tinea manuum. This case also underscores the importance of advanced diagnostic methods for accurate fungal identification, including PCR, sequencing, and mass spectrometry.
